# Moving Forward from Moral Injury: A Mixed Methods Study Investigating the Use of 3MDR for Treatment-Resistant PTSD

**DOI:** 10.3390/ijerph20075415

**Published:** 2023-04-06

**Authors:** Lorraine Smith-MacDonald, Chelsea Jones, Matthew R. G. Brown, Rachel S. Dunleavy, Annelies VanderLaan, Zornitsa Kaneva, Tristin Hamilton, Lisa Burback, Eric Vermetten, Suzette Brémault-Phillips

**Affiliations:** 1 Heroes in Mind, Advocacy and Research Consortium (HiMARC), Faculty of Rehabilitation, University of Alberta, Edmonton, AB T6G 2G4, Canada; 2 Faculty of Rehabilitation Medicine, University of Alberta, Edmonton, AB T6G 2G4, Canada; 3 St. Stephen’s College, Edmonton, AB T6G 2J6, Canada; 4 Alberta Health Services, Edmonton, AB T5J 3E4, Canada; 5 Department of Computing Science, University of Alberta, Edmonton, AB T6G 2S4, Canada; 6 Department of Psychiatry, University of Alberta, Edmonton, AB T6G 2B7, Canada; 7 Department of Psychiatry, Leiden University Medical Centre, Leiden University, 2333 ZA Leiden, The Netherlands

**Keywords:** moral injury, military, veteran, treatment-resistant PTSD, 3MDR, virtual reality

## Abstract

Background: Exposure to trauma and potentially morally injurious events may lead to moral injury (MI). The link between MI and posttraumatic stress disorder (PTSD) may have particularly relevant implications for treatment-resistant PTSD (TR-PTSD). Multi-modal Motion-Assisted Memory Desensitization and Reconsolidation (3MDR), a technology-assisted exposure-based trauma therapy that has been used in the treatment of PTSD, may also be an acceptable modality for patients in the treatment of TR-PTSD and MI. This proof-of-concept study aimed to investigate (1) whether MI co-occurs in military members (MMs) and veterans with TR-PTSD, and (2) the perspectives of MMs and veterans with TR-PTSD utilizing 3MDR for MI. Methods: This study employed a mixed-methods clinical trial. Military Members and veterans participated in this study (N = 11) through self-reported questionnaires, video recordings of treatment sessions, and semi-structured interviews post-session and post-intervention, with longitudinal follow-up to 6 months. Results: MI scores correlated with self-reported measures of mental health symptoms related to PTSD. The thematic analysis revealed three emergent themes: (1) Realities of War, (2) Wrestling Scruples, and (3) Moral Sensemaking. Conclusion: MI was highly correlated with TR-PTSD and themes regarding MI. This result, while preliminary, allows for the postulation that MI may be contributing to the continuation of PTSD symptoms in TR-PTSD, and that 3MDR may be an acceptable modality for addressing these symptoms in MMs and veterans.

## 1. Introduction

Military members (MMs) and veterans are exposed to experiences which often include traumatic elements, large scale human suffering, and the transgression of universal human morals. The potential harm caused by exposure to such traumatic experiences was recognized by the American Psychiatric Association (1980) with the introduction of the posttraumatic stress disorder (PTSD) diagnosis in the Diagnostic and Statistical Manual of Mental Disorders-III (DSM-III) [1]. Largely underexplored until recently, however, has been the harm caused by exposure to human suffering and moral transgressions. Within military literature, the introduction of the term “moral injury” (MI), first used to describe Vietnam veterans [2] and then more recently to describe post-911 veterans [3], has illustrated that trauma alone may not fully encapsulate the harm that soldiers may experience because of the environment and nature of their work (e.g., combat, peacekeeping mission). Used to describe the persistent distress that individuals may develop when they perpetrate, witness, or fail to prevent an act that transgresses their core beliefs [3], MI is increasingly being referred to as a syndrome characterized by widespread psychological, emotional, social, and spiritual impairment [4].

Exposure to potentially morally injurious events (pMIEs) has been discussed as the precursor leading to MI [5]. In the context of war, pMIEs have been associated with “participating in or witnessing inhumane or cruel actions, failing to prevent the immoral acts of others… engaging in subtle acts or experiencing reactions that, upon reflection, transgress a moral code [or] bearing witness to the aftermath of violence and human carnage” [3]. There is considerable debate as to whether or not a pMIE “must be’’ an event that would meet Criterion A for PTSD according to the DSM-5 [6].

It is possible that “moral dilemmas” can exist in the absence of trauma. Studies of military personnel and veterans have found that a significant minority of index traumas for PTSD are events that do not primarily evoke fear/threat [7]. In addition, MIs that are index traumas for PTSD are more strongly associated with emotions that develop after the event rather than emotions experienced during it (e.g., intense feelings of guilt and shame) [8]. The underlying mechanism leading to the development of MI may instead be how the individual appraises and assigns meaning to the event rather than the event itself [9]. MI may also have unique complex pathophysiology which may either align, overlap, or be completely different from that of PTSD [10]. Nonetheless, MI has been noted to frequently occur comorbidly with PTSD, major depressive disorder, generalized anxiety disorder, and substance use disorder, along with other psychosocial challenges such as self-harming or risky behaviors, social isolation and alienation, and fractures with one’s relationship to self, others, the world, and the transcendent [11,12,13].

The possible link between MI and PTSD may have particularly relevant implications for emerging discussions of treatment-resistant PTSD (TR-PTSD). The classification of TR-PTSD has been adopted for the many MMs and veterans who do not experience a clinically significant reduction in symptoms following receipt of at least two evidence-based treatments [14,15]. Knowledge of TR-PTSD is limited and only general treatment recommendations for TR-PTSD have been suggested. Specific protocols or evidence-based TR-PTSD therapies are lacking, complicating clinical attempts to address or manage this condition [16]. Given the potential for MI to be a negative component or compounding factor of PTSD, MI may be all the more associated with TR-PTSD. For example, unresolved MI may support the continuation of PTSD symptoms despite the use of evidence-based PTSD treatments [17]. The fact that military-related trauma may not be fully explained by a fear-based paradigm may support this idea, especially since veterans do not respond as well to existing evidence-based PTSD interventions compared to civilians [18,19]. As such, novel treatments are needed to address PTSD, TR-PTSD, and MI.

### Multi-modal Motion-Assisted Memory Desensitization and Reconsolidation (3MDR)

Evidence-based treatments for TR-PTSD and MI are needed to assist in the rehabilitation and recovery of trauma-affected populations. Multi-modal Motion-Assisted Memory Desensitization and Reconsolidation (3MDR) is an innovative, technology-assisted exposure-based trauma-therapy that holds promise for treating both TR-PTSD and MI. 3MDR is an emerging therapy delivered in an immersive virtual reality (VR) system that includes a treadmill and large format visual display. Previous 3MDR studies have used a Computer Assisted Rehabilitation ENvironment (CAREN) system - a room-sized, 3-dimensional, VR-system with a central treadmill, surrounded by 240-degree floor-to-ceiling motion-capture screens [20].

During each of six 90-minute 3MDR sessions, participants continually walking on the treadmill with a clinician standing beside who guides them through 3MDR’s three phases: (A) a *pre-platform phase* to select and order symbolic representations and identify music; (B) *a platform phase* involving a warm-up of walking while listening to music, the viewing of 7 images for 3-5 minutes each, and a cool-down of walking while listening to music [21,22]. The participant describes each image and associated traumatic events or pMIEs, together with physical sensations, emotions, and thoughts that arise. As in Eye-Movement Desensitization and Reconsolidation therapy (EMDR), a virtual ball displaying numbers (which the participant reads out loud) then moves back and forth across the screen in the foreground of the image before the next image appears. (C) *A post-platform phase* provides an opportunity for review, discussion of new insights, and a self-care plan. 

Several key elements of 3MDR therapy have been associated with reduced PTSD symptoms. These include: (1) engaging in exposure to traumatic material whereby avoidance patterns are minimized or broken; (2) experiencing and giving expression to emotions in the here and now that are evoked while the traumatic memories are being retrieved; and (3) reintegrating (reconsolidating) memories and sensory stimuli along with affective information associated with the trauma via a dual-attention task. It is thought that these elements combined work collectively to facilitate the memory reconsolidation necessary to reduce the intensity of traumatic memories and subsequent PTSD symptoms [22]. Jointly, exposure to VR visual imagery and auditory input, walking, the dual-attention task, and the therapeutic context and relationship may support 3MDR effectiveness. Multiple studies utilizing 3MDR within military and veteran populations with TR-PTSD in the Netherlands, United Kingdom, United States, and Canada have demonstrated promise [23,24,25,26]. Decreases in PTSD symptom severity from baseline to the trial endpoint were significantly greater for the 3MDR group compared to the control group, with medium to large effect sizes [23,24]. The impact of 3MDR on symptoms of PTSD and related conditions continues to be explored.

The effectiveness of 3MDR in addressing MI has yet to be studied. 3MDR may be a valuable treatment option for addressing MI because it makes avoidance of traumatic stimuli or PMIEs challenging. Research has indicated that it is often difficult for persons with MI to share about their PMIEs both within and outside of therapeutic settings [27]. Therefore, treatments, such as 3MDR, that do not allow MMs and veterans to hide or minimize PMIEs or MI may be a key to the recovery from PTSD and MI. Moreover, with a strong focus on emotions, 3MDR may be a more viable treatment for MI than more cognitively based treatments (i.e., Cognitive Processing Therapy or Cognitive Behavioral Therapy) as MI has historically been defined by the emotions of guilt and shame [3]. More targeted emotionally-focused MI treatment can address the broader spectrum of moral emotions (i.e., anger, rage, despair, contempt, disgust, shock, awe, etc.) [28]. As 3MDR requires participants to identify emotionally triggering phrases that can be displayed on the screen and read aloud, this may mirror narrative practices of lament [29] and expressive writing [30], both of which have been suggested to be useful in the treatment of MI. 

Unlike most evidence-based trauma-therapies, 3MDR combines movement with exposure and narrative. Physically moving forward during exposure to emotional memories may open the door to new learning and memory reconsolidation leading to mental shifts around themes of guilt, shame, and self-blame [31]. This movement during therapy, combined with a reduction in the ability for the participant to cognitively avoid traumatic stimuli, may enhance divergent thinking abilities by stimulating hippocampal activity and allowing the patient to change patterns of problematic thinking and reduce cognitive rigidity [31]. Several peer-reviewed articles further describe the 3MDR intervention and speak to these factors [24,32]. The concurrent ability of 3MDR to potentially treat both PTSD and MI may be unique to this treatment approach and a potential reason for its efficacy.

The purpose of this proof-of-concept uniquely aimed to investigate (1) whether MI co-occurs in military members and veterans with TR-PTSD, and (2) the perspectives of military members and veterans with TR-PTSD on utilizing 3MDR for the treatment of MI. Although the naming of the phenomenon of MI occurred relatively recently, aspects of MI have been recognized since the earliest literature on PTSD. As a result, it was hypothesized that MI would be both associated with TR-PTSD experienced by the participants and present in the data from the 3MDR intervention.

## 2. Materials and Methods

### 2.1. Study Design

This mixed methods randomized controlled trial employed a waitlist crossover design. A previously published protocol paper describes the methods of this study in detail [32]. The current manuscript represents the pilot data of the first 11 participants who completed the aforementioned study protocol [32], including follow-up data collection up to 6 months post-treatment. All 11 participants included in this manuscript were part of the intervention and not the control group. While a waitlist control group was originally planned, COVID-19 related measures halted data collection before waitlist control data could be collected. This study has been approved by the University Health Research Ethics Board (Pro00084466) and received endorsement from the Canadian Armed Forces Surgeon General Health Research Program (E2019-02-250-003-0003).

### 2.2. Sample and Setting

A sample of regular Canadian Armed Forces (CAF) MMs and veterans were recruited for the study using local sources. This included recruiting from CAF medical clinics and operational stress injury clinics in addition to using snowball sampling (i.e., word of mouth). The study’s *a priori* inclusion criterion required that participants be English-speaking, aged 18–60 years, have experienced trauma in a military combat setting, be able to ambulate safely on a treadmill for at least 45 min, and meet the DSM-5 criteria for a diagnosis of PTSD. All participants had a score of 30 or higher on the Clinician-Administered PTSD Scale for DSM-5 (CAPS-5) Worst Month version. Participants were classified as having combat-related TR-PTSD, indicating they had previously not responded to at least two types of evidence-based treatments, at least one of which was a psychotherapeutic intervention. Participants were stable on their current psychotropic medication for at least 4 weeks before entering the study. Individuals with co-morbidity were included if they satisfied the other inclusion/exclusion criteria. Potential participants were also screened by a member of the research team based on military employment/deployments, current and past medical history, history and experiences of previous PTSD interventions, and overall suitability before providing verbal and written consent to participate. Participants who did not match the above inclusion criteria were excluded from the study. The delivery of the 3MDR intervention occurred in a rehabilitation hospital in Western Canada.

### 2.3. Sample Size

A sample size calculation was conducted using G*Power 3. To detect a significant interaction with at least a medium effect size, a minimal sample size of 17 participants was determined for each group. Because of an estimated 10% dropout and some attrition at measurements, the sample size was set at 20 participants in each group (Cohen f = 1.0) using general linear modeling with one within-subjects factor (two-time points) and one between-subjects factor (two interventions), and assuming a correlation of r = 0.5 between the repeated measures, an α-level of 0.05, and a power. Due to the COVID-19-related measures, it was necessary to end the planned data collection prematurely. Of 17 recruited participants, 4 were unable to complete the 3MDR intervention due to the COVID-19 measures. Two participants who completed the 3MDR therapy opted out of the study before completing follow-up data collection. The remaining participants (n = 11) completed 3MDR and subsequent follow-up measures up to 6 months post-intervention. Data from this sample was completed. Despite not reaching our initial sample size, the effect sizes were large enough to allow our analyses to detect statistically significant effects (see Results).

### 2.4. Intervention

For this current study, the intervention consisted of six sessions of 3MDR received once a week. Conversely, control participants received TAU through their mental health providers or organizations while they were waiting for the intervention. The definition of TAU for the purpose of this study included evidence-based psychotherapeutic interventions such as CBT, CPT, PE, EMDR, and pharmaceutical interventions [14,15].

### 2.5. Data Collection

Data were collected before treatment commenced, after treatment was completed, and at follow-up sessions of 1 month, 3 months, and 6 months post-intervention. We will refer to these time points as pre, post, 1 month, 3 months, and 6 months, respectively.

### 2.6. Quantitative Data

A series of self-report questionnaires were collected, at time points pre, post, 1 month, 3 months, and 6 months, in order to measure changes in a number of constructs related to PTSD and MI. These included The Military Injury Symptom Scale–Military Short Form (MISS-M-SF) [33], the Clinically Administered PTSD Scale for DSM-5 (CAPS-5) [34], the PTSD Checklist for DSM-5 (PCL-5) [35], the Patient Health Questionnaire (PHQ-9) [36], the Generalized Anxiety Disorder Scale (GAD-7) [37], the Outcome Questionnaire 45 (OQ-45.2) [38], the Dissociative Experiences Questionnaire (PDE-Q) [39], the CD-RISC-25 (Connor-Davidson Resilience Scale) [40], the Alcohol Use Disorder Identification Test (AUDIT) [41], and the Difficulties in Emotion Regulation Scale (DERS-18; 6-months data was not available) [42]. A description of the included outcome measures follows.

The Military Injury Symptom Scale–Military Short Form (MISS-M-SF) was the primary outcome measure of interest for this study. The MISS-M-SF is a reliable and valid measure of MI symptoms that can be used to screen for MI and monitor response to treatment in veterans and active-duty military with or without diagnosed PTSD [33]. The possible range of scores is from 10 to 100. The total score is an indication of functional impairment caused by MI.

The CAPS-5 is a 29-item structured interview for assessing PTSD diagnostic status and symptom severity [34]. The CAPS-5 is the gold standard in PTSD assessment and can be used to make a current (past month) or lifetime diagnosis of PTSD or to assess symptoms over the previous week. The items correspond to the DSM5 criteria for PTSD.

The PCL-5 is a widely-used, 17-item questionnaire self-report measure of PTSD symptom severity in relation to an identified “stressful experience”. Each symptom is rated on a scale of 0–4. This approach has been used before and has strong reliability and validity [35].

The PHQ-9 is commonly used to measure the severity of depression. The PHQ-9 incorporates DSM-IV depression diagnostic criteria into a 9-item, self-report outcome measure. Responses represent the frequency of symptoms in the past two weeks, and each symptom can be rated on a scale of 0–3 [36]. A score between 5–9 indicates mild depression; 10–14 indicates moderate depression; 15–19 indicates moderately severe depression; and 20–27 indicates severe depression [36].

The GAD-7 is used to measure the severity of anxiety. This self-report scale consists of seven items, and responses represent the frequency of symptoms in the two past weeks and are given on a scale of 0–3 [37]. Scores between 5–9 indicate mild anxiety; 10–14 indicate moderate anxiety; and 15–21 indicate severe anxiety [37].

The OQ-45 is a self-report inventory measuring social functioning. It consists of 45 items which are rated on a 5-point scale and reflect three domains: symptomatic distress, interpersonal relationships, and social role [38]. A total score of 63 or more indicates symptoms of clinical significance, and a difference of 14 points or more (between sessions) indicates a significant change in symptoms [38].

The PDE-Q is a 10-item test that measures the extent of dissociation at the time of the traumatic event and in the minutes and hours that followed [39]. Studies suggest that dissociation increases the risk of developing PTSD [39]. PDE-Q administration and scoring take under 5 min each. All items are scored from 1 (not at all true) to 5 (extremely true), and the total score is the sum of all items [39]. A score above 15 is indicative of significant dissociation [39].

The CD-RISC-25 is a tool utilized to measure perceived resilience within 17 domains. The tool consists of a 25-item scale within these domains. This tool has been studied extensively and has been demonstrated to be valid and reliable when utilized with survivors of various traumas and PTSD [40].

The AUDIT is an alcohol self-report, 10-item questionnaire that aims to help identify persons who are hazardous drinkers or have active alcohol use disorders [41]. A score of 8 or more is considered to indicate hazardous or harmful alcohol use [41].

The DERS-18 is a 36-item self-report measure that aims to assess emotion dysregulation. It has been translated into several languages; short forms have been developed including the 18-item DERS-18 [42]. Items are rated on a scale of 1 (“almost never [0–10%]”) to 5 (“almost always [91–100%]”). Higher scores indicate more difficulty in emotion regulation [43].

### 2.7. Qualitative Data

The qualitative data consisted of portions of the video recordings of the six weekly treatment sessions, the audio-recorded debriefs after each of the six weekly treatment sessions, and semi-structured interviews conducted 1 month, 3 months, and 6 months post- intervention. Examples of the types of questions asked of study participant during the semi-structured interviews included but were not limited to (1) what is your experience of 3MDR?; (2) How did the intervention impact you?; (3) What elements of the intervention were the most helpful/impactful/effective?; and (4) What elements of the intervention were the least helpful/impactful/effective?

### 2.8. Data Analysis and Triangulation

A mixed-methods research design allows for meaningful data analysis and interpretation of MI as it relates to 3MDR. While the quantitative data may provide insight into whether MI is present, the qualitative data will provide information regarding the presence of MI and further explore why and how MI is experienced. A concurrent parallel approach following a data transformation model was utilized for data triangulation [44]. Converging data allowed the research team to compare and contrast quantitative and qualitative data and support subsequent study design, data collection, and analysis.

### 2.9. Quantitative Analysis

We examined the relationship between moral injury MISS-M-SF score values and 20 different scores derived from the aforementioned outcome measures and their constructs. This included scores from the PCL-5, PHQ-9, GAD-7, OQ-45.2, PDE-Q, CD-RISC-25, AUDIT, CAPS-5, (total symptoms, B: re-experiencing, C: avoidance, D: negative alterations, E: hyperarousal, and dissociation), and DERS-18 (awareness, clarity, goals, impulse, non-acceptance, and strategies).

For each score, a linear model was used to estimate the relationship between MISS-M-SF score values and the values for the given score. The linear model included a term for MISS-M-SF score values (i.e., concatenation of the 11 mean-centered MISS-M-SF score time courses, one-time course from each participant) as well as 11 offset terms, one for each participant. For a given score, the data used to fit the model was derived by concatenating the score time courses from each of the 11 participants. The linear model was fitted to the data using least squares, and the fitted parameter for the MISS-M-SF term provided an estimate of the relationship between MISS-M-SF score values and a given score.

Due to logistical complexities around COVID-19, some participants did not contribute data to certain time points for certain instruments. Across all analyses performed, 84% of data points were present. That is, across all combinations of instruments (questionnaire or interview), participants, and timepoints, 16% of data points were missing. The statistical model included offset terms for each participant to account for between-subject differences in mean scores. The model was therefore able to regress out any confounding time-related changes (if such were present) created by possible first-order interaction between the pattern of missing data and between-subject differences in mean scores. The model statistically tested aggregates within subject time-related changes in scores.

*p*-values were computed using permutation testing. Permutation testing is non-parametric and was chosen because it does not make assumptions about the shape of the data distribution. For a given score, an empirical distribution with 100,000 samples was generated by randomly permuting the score values at different time points within each participant and then fitting the linear model as described above. A total of 99,999 iterations of permutation and fitting were conducted for each score. The resulting fitted parameter values for the MISS-M-SF term comprised the empirical distribution. The actual fitted MISS-M-SF term parameter value was also included as the final sample in the empirical distribution, as is standard practice. The actual fitted MISS-M-SF term parameter was compared against the empirical distribution to generate a *p*-value.

A total of 20 statistical tests were performed. The Benjamini-Hochberg [45] procedure for false discovery rate (FDR) correction was used to handle multiple comparisons.

### 2.10. Qualitative Measures

The qualitative data were recorded, transcribed, and thematically analyzed. Braun and Clarke (2006) described thematic analysis as a method for identifying, analyzing, and reporting patterns (themes) in rich detail, which when done properly may also allow for the research to interpret various aspects of the phenomena in question. Practically, thematic analysis involves examining the text in detail to identify recurring patterns (open coding), which are refined into themes through inductive and/or deductive analysis [46]. That is, those that arise directly from the data and those which relate to theory and previous findings, respectively. For this study, inductive coding was used to examine participant experiences and an understanding of MI without preconceived notions, while deductive coding was used to answer the research questions.

Data analysis began with research team members reading and/or listening to all of the qualitative data, followed by six members of the research team (two senior and four junior) developing preliminary open codes. Once open codes had been established, each participant’s file was then reviewed by another member of the team to support rigor and reliability. After validating the codes, the core coding research team began combining the codes into preliminary themes. These preliminary themes were then analyzed and verified by the larger research team as an extra measure of rigor, with any differences being resolved through ongoing discussion among all authors. Having established the preliminary themes, an initial draft of the proposed thematic framework was written. The thematic framework underwent a second round of collective analysis, with the preliminary themes being modified to reflect a more nuanced and richer understanding of the data. Participant quotes, illustrating each sub-theme, were selected for their representativeness and incidences of divergent opinions. Despite the small sample size, data saturation – the point at which no new data was found—was reached. It became clear within the first 5–7 participants (using all their data time points) that there were coherent themes regarding the potential use of 3MDR to treat MI across participants.

Following the concurrent parallel approach, the quantitative and qualitative data were triangulated and compared to formulate the results and inform the future of the study.

## 3. Results

### 3.1. Demographics

Demographics for the 11 participants are shown in Table 1. All participants were either active CAF MMs or veterans, and all of them were deployed to an active military theater at least once in their careers.

### 3.2. Quantitative Results

**Figure 1:**
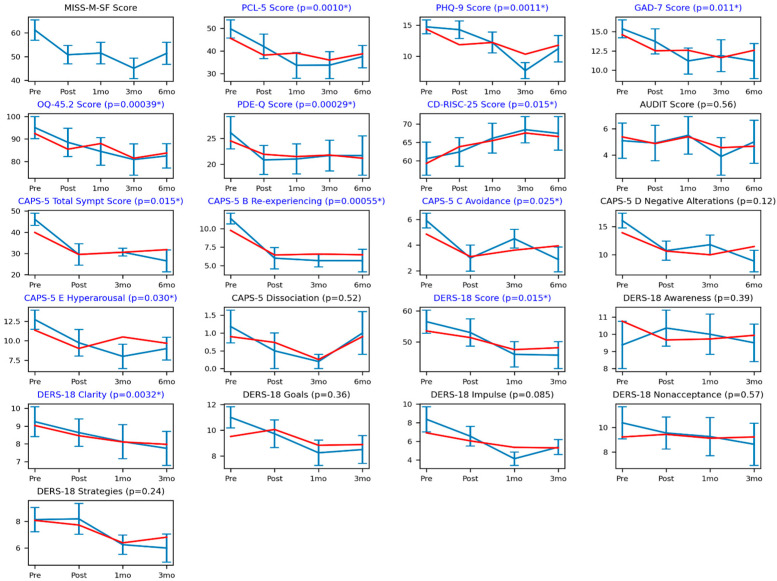
The blue lines show the average scores across the 11 participants at each time point. Error bars indicate the standard error of the mean. The red lines are the estimated scores over time generated from a statistical model that “explained” changes in a given score in terms of the moral injury MISS-M-SF scores. *p*-values in brackets were generated from these statistical models based on the strength of the relationship between the MISS-M-SF scores and the given score being tested. An asterisk indicates that the *p*-value was significant and survived FDR multiple comparison correction (the *p*-value threshold was computed to be 0.030). In addition, graphs for which the *p*-value was significant and survived multiple comparison corrections have a blue title (as opposed to the black title for non-significant results).

We examined statistical relationships, as presented in Figure 1, between changes over time in the MISS-M-SF scale and in scores from the other questionnaires or from the CAPS-5 semi-structured interview. Specifically, for a given score, we fitted a linear model including the participants’ MISS-M-SF scores as a predictor term to the score values. We tested the fitted weight for the MISS-M-SF predictor against a null hypothesis value of zero using permutation testing. MISS-M-SF scores were statistically significantly related to the PCL-5 score, PHQ-8 score, GAD-7 score, OQ-45.2 score, PDE-Q score, CD-RISC-25 score, CAPS-5 Total Symptom score, CAPS-5 B Re-experiencing score, CAPS-5 C Avoidance score, CAPS-5 E Hyperarousal score, DERS-18 overall score, and DERS-18 Clarity score. These results survived FDR multiple comparison correction; the computed FDR *p*-value threshold was 0.030. Measures that did not show a significant relationship with MISS-M-SF scores included the AUDIT, two of six scores from the CAPS-5 (Negative Alterations and Dissociation), and five of seven scores from the DERS-18 (Awareness, Goals, Impulse, Nonacceptance, Strategies).

### 3.3. Qualitative Results

Inductive and deductive thematic analysis of the qualitative data found three themes that emerged: (1) Realities of War, (2) Wrestling Scruples, and (3) Moral Sensemaking.

#### 3.3.1. Realities of War

Participants engaging in 3MDR reported feeling broken and not understanding why previous treatments had not been able to address this overall state. Early in 3MDR treatment, participants expressed that many of the realities of war or their deployment experiences were unresolved or incomplete. For example, participants noted that, once deployed, their experiences were nothing like they had expected, and they had to reconcile the decisions they made or followed. Participants often found themselves putting their heads down, following orders, and putting mission over morals because it was their job; deviating from their role could put their comrades, troops, friends, and civilians at risk. However, despite intellectually acquiescing to their duty to put the mission first, participants reported feeling helpless, hopeless, confused, jaded, disempowered, and angry. In response to this distress, participants noted creating a “black box”– an intrapsychic place where soldiers file responses and reactions (psychological, emotional, behavioral) which could infringe on their ability to do their job. While distinct and uniquely personal, participants noted common narratives which were often rooted in burying conflicting emotions- and values-based accounts about deployment experiences. More simply, the contents of their black boxes were a tangled mess of deeply personal, unpleasant emotions and conflicting beliefs and narratives about themselves, their identity as a soldier, the military, and the world in general.

*“First time out, you know, everyone was smiling, we were having a good time and by the end of the tour, you’re basically threatening to kill kids.” *

*(P13)*

*One of the pictures I have over there is a painting of what they would do with the children, they would skeet shoot them, throw them in the air and see if they could hit them with bayonets, things like that. It was a little troubling [laughing] *

*(P10)*

*“If you can’t dump it, you gotta file that in the back of our head, because there is still a mission that needs to go on and nobody wants to talk about that shit, ok? So, (…) unfortunately, sometimes that [door] gets cracked open and I could never shut that door completely, I could never file completely in the back of my mind to make sure that I could get rid of it.” *

*(P19)*

*I couldn’t do anything because I wasn’t allowed to do anything. I didn’t do what I wanted to. It doesn’t make it okay.” *

*(P11)*

*“How do I go and do this [mission] when I know it is wrong? Like, morally it’s wrong. Ethically, it’s wrong. But I’m told I gotta do this. What do you do? How do you function with that? And you know, I don’t mean squeezing the trigger. I don’t mean lobbing artillery. I don’t mean driving over stuff. It’s the fact of, people are hurt and they need help, there are good people and bad people.” *

*(P19)*

*“[I felt] abandoned… When there were a lot of us, after those two weeks [spent cleaning up after the traumatic event], we were just cut loose. We were given one day off then told to go back [to regular duties]…just by the organization in general.” *

*(P6)*

#### 3.3.2. Wrestling Scruples

During 3MDR, participants’ narratives offered insight into trauma-related symptoms that differed from PTSD criteria. While they may have experienced significant traumatic events during their time during or after service, these experiences were not always the most injurious or may not have directly resulted in an MI. A melding of continuous and conflicting experiences, an inability to resolve their distress, and a perceived lack of support through their career culminated in MI. While guilt and shame, the proposed signature symptoms of MI, were present, significant other emotions such as horror, contempt, disgust, rage, bitterness, numbness, despair, disappointment, worthlessness, regret, shock, disillusionment, and confusion were also often comorbid. Participants reported feeling stuck and alone in this liminal cognitive and existential nightmare, constantly questioning their worldview, beliefs, actions, moral compass, and selves. Central to this theme was an unresolved dissonance between what participants perceived as “ought ‘‘ to have happened versus what truly “did” happen.

*“But we don’t understand that those that are left behind carry the pain, it stays with them, that we feel nothing, barren…” *

*(P13)*

*“I felt trapped in my little existence, I guess, like, shit… This is the way it’s going to be from now until whenever it ended you know. And that was scary…very depressing. Like trapped. I was stuck there… I’m going to have to put up with this all the time. And eventually, it’s going to crush me.” *

*(P6)*

*“Why did you do this? Like why couldn’t you get along? I was always told, ‘work things out, trust people…’ All I saw was the good and never the bad …My whole life got shattered right there and then. All I wrote about when I came back was the devastation of what I saw. I did feel devastated. I felt the heartless[ness] of people.” *

*(P3)*

*“This feeling of being lost really. Not understanding, not being able to comprehend… comprehend what happened.” *

*(P7)*

*The word shame is … a … Huge. You take pride in everything you have accomplished. When you don’t live up to that persona, even in your own mind or even to those around you, it’s… it’s painful.” *

*(P13)*

*I’m worried that it means we were a little less than human and more mechanical in what we did. It was all mechanical… you follow the checklist; you do what you’re trained to do. Could we have done more? The honest answer is no, but we don’t know.” *

*(P19)*

*“Every time the kids fight, and someone gets hurt, I just see the crying face of this little girl looking up at me and not knowing if I’m there to help or hurt. Just so little and dependent on everyone around her to help (…) I’m not looking at my grandson or kids like they’re this little girl, but it automatically takes me back to this coppery smell in the air, all that blood, and then the eyes.” *

*(P19)*

#### 3.3.3. Moral Sensemaking

During 3MDR, participants began to make sense of their own experiences, choices, and actions, and reconcile with who they felt they had become. In their sensemaking quest, participants tackled big questions and faced the chaos and demons in their Black Box with honesty and humility. That is, they felt supported and thus were able to become vulnerable through identifying and giving voice to unfettered thoughts and feelings, rather than remaining on autopilot or repeating previous narratives. They were able to lament without being judged by allowing themselves to share their messy truths. Many reported a shift from an ideological either/or worldview to a multifaceted worldview that accepted rather than rejected their emotional reactions, dissonant cognitions and fractured worldviews. Through this messy, painful process, participants were able to make sense of their unconscious experiences, reconcile their value-based pain, and (re)claim their self-dignity. They were also able to feel the full spectrum of emotions related to their deployment(s) experiences, their actions, the world, and themselves.

*[3MDR] showed me how not to suppress the emotional aspect, but just come to grips with it (…). How you feel is how you feel. There is nothing wrong with feeling that way, but understand why you feel that way.” *

*(P19)*

*“Weight on my neck, stopping me from doing what I need to do with my kids—guilt, pain, sorry, the feeling that I might actually be human. I might have emotions aside from what I’ve been trained to deal with.” *

*(P2)*

*“The pain that I didn’t know was there… Never even considered… I buried it so deep that I just left it where I put it. And I open that box. Once the box is open, it basically gets poured out. And either… you can make the decision to either fill the box back up or just leave it on the ground. And I think I have left it on the ground.” *

*(P13)*

*Maybe I was just there to give somebody a little bit of dignity in death. And I think that kind of helped [me].” *

*(P6)*

*“Stuff still gets under my skin; I’m human. I still have reactions, I just don’t blow up about it.” *

*(P19)*

*“Started to realize there is good and bad everywhere, no matter where you go.” *

*(P2)*

*“Well I’m saying goodbye to my, my.. demon and my…shame; my perceived shame [strong emphasis]” *

*(P13)*

*“I was very closed, I was very closed off; primarily to my family, but you know, to everyone I was very closed. I never wanted to say anything because you know what, I would stay open. [broken up] Everyone has parts of their own pasts that they have to deal with. Who am I to dump more crap on someone’s head? Right? Unfortunately, what wound up happening was me trying to internalize it was tearing me apart.” *

*(P19)*

## 4. Discussion

The purpose of this proof-of-concept study was to investigate (1) whether MI co-occurs in MMs and veterans with TR-PTSD, and (2) the perspectives of MMs and veterans with TR-PTSD in utilizing 3MDR for the treatment of MI. It was hypothesized that 3MDR would be an acceptable modality for addressing TR-PTSD within MMs and veterans, and that MI would correlate with the TR-PTSD experienced by the participants. Triangulation of both data sets supported the hypothesis that MI was co-occurring in MMs and veterans with TR-PTSD. Quantitatively, these results illustrated that MISS-M-SF scores were statistically significantly related to scores of PTSD, major depressive disorder (MDD), generalized anxiety disorder (GAD), peritraumatic dissociation, emotional regulation, interpersonal relationships and social role, and resilience. While the research to date has repeatedly shown a strong association between MI, PTSD, MDD, and GAD [5,47,48], this is the first study to illustrate an association between MI and TR-PTSD. This result, while preliminary, does allow for the postulation that MI may be contributing to the continuation of the PTSD symptoms in TR-PTSD. 

Given the growing body of literature illustrating that MI is correlated with PTSD, GAD, and MDD, it may be helpful to begin a transdiagnostic exploration of the mechanisms driving the pathologies of these from both an MI perspective and a psychiatric disorder perspective. The question of dissociation and MI, for example, has not been addressed to date, and therefore it is unclear in what ways MI may be contributing to dissociation symptoms or vice-versa. This raises some interesting questions about if and how MI may be related to dissociative-type PTSD. In this study, the CAPS-5 dissociation score’s association with the MI score was not statistically significant; however, the PDE-Q did show a statistically significant association. Dissociation was not mentioned in the qualitative results despite the discussion of extreme emotional responses. Some research has identified that intense shame, guilt, or psychological conflict can push people into a dissociative state [49,50,51]. Given this, it may be possible that people with increased trait dissociation, a noted potential marker of earlier or complex trauma [52], are more likely to develop MI when they face pMIEs. This may particularly be the case when shame is associated with the pMIE or MI [53].

Regarding a potential relationship between MI and alcohol consumption, scores from the AUDIT did not show a significant relationship with MI scores. The AUDIT is most sensitive to severe alcohol consumption, as would be seen with a substance use disorder. We suspect that the AUDIT was not sufficiently sensitive to changes in alcohol consumption falling in a more moderate range, as exhibited by the study participants, to detect any changes that might have occurred.

Questions related to the role of emotional (dys)regulation in MI and PTSD are also found in these results. Previous literature has highlighted the potential role of emotional dysregulation in PTSD [54,55], especially in military and veteran populations [56]. As such, the potential of emotional (dys)regulation to be relevant to TR-PTSD also seems plausible. Equally, preliminary research on emotional regulation and MI to date has shown mixed results. Previous qualitative research has noted that veterans suffering from MI often experience negative emotions [57,58]. While a recent study suggests that the severity of MI is positively correlated with the severity of Cluster C and D PTSD symptoms, emotional dysregulation interestingly was not correlated with MI [59]. Our quantitative results support aspects of the above studies in that Criterion D was not statistically significant, while Criterion C was. Curiously, negative alterations did not change given the focus of MI research on emotions such as guilt and shame, and the potential negative change in assumptive worldviews. Our qualitative results did show the potential role of negative alternations in both mood and cognition as being related to both MI and PTSD. Further research is required to understand the role of emotions in MI and the potential complement or difference between those feelings expressed in MI and those expressed in TR-PTSD.

The quantitative results also demonstrate an association between the overall DER-18 score and the MISS-M-SF. Only one of the six subscores—clarity—however, was significant. Again, while the reasons for this cannot be presumed, it may be that in processing cognitions and symptoms, participants were able to learn, identify and clarify their own emotions. Qualitatively, participants also spoke of the importance of emotions in the maintenance of their PTSD and MI, and the power of being able to label and give a voice to emotions during and after their traumatic experiences.

While guilt and shame have been the signature emotions associated with MI, our participants also experienced a range of moral emotions [29], such as rage, contempt, disgust, regret, disillusionment, and despair. A narrow focus on the emotions of guilt and shame may, therefore, not fully encapsulate the realities of MI. This also differs from the Cluster D emotions found within the DSM-5 diagnosis for PTSD which focus on exaggerated blame [6]. Moreover, there may be tension or dissonance from experiencing moral emotions such as pride and honor simultaneously with those such as disgust and regret. Screening related to MI must, as a result, be holistic and purposely explore contradictory or opposite emotions. Care must also be taken not to overstate this relationship nor deduce causality. Further research is needed to deduce the potential role of emotional (dsy)regulation for both TR-PTSD and MI.

The potential importance of MI to combat-related TR-PTSD is notable and appears deeply woven in the fabric of this qualitative analysis. Participants’ self-disclosed qualitative experiences illustrated that while they were not always aware or fully able to articulate the nature, harm and scope of what happened to them, they were aware that harm had occurred at a deeper and fundamental level. These results support the notion that MI seemingly fractures a person at the level of “self” [60,61], which, if left buried in the black box, can result in a slow disintegration of self, one’s career and life, and the ability to engage in the world. Central to this harm was the inability to contain the distress of conflicted emotion-, value-, and morally-laden experiences. These results pose an interesting difference between PTSD symptoms and those of MI, as the focus of these morally-laden experiences were not always fear-based (i.e., Criterion A). This was seen especially in theme two, Wrestling Scruples, where the focus of the therapy was to allow participants to give voice to and acknowledge the tension between what “ought ‘‘ to have happened versus the reality of what “did happen” [29]. Importantly, this scrupulous “is”/“ought” dichotomy was experienced by participants not as cognitive dissonance (i.e., Cluster C and D symptoms), but as a type of ontological dissonance (i.e., a dissonance in "who I am" and how/who do I want to *be in the world)*. The association between the MISS-M-SF and nearly all of the measured outcomes illustrate the pervasive ontological nature of MI within the context of trauma.

Based on these preliminary results, it is theorized that MI may fundamentally impact the ability to trust, resulting in fragmented relationships and poor mental health outcomes [62]. Such a theory may help to explain why addressing MI in 3MDR allowed the participants to experience a change in their OQ-45 scores, which measured perceived social functioning. Preliminary research has noted that there may be a bidirectional relationship between MI and social support, in which social support may both reduce the symptoms of MI, and be one of the key protective factors that becomes impeded when someone is morally injured [63,64]. For example, Koenig et al., (2018a) observed strong inverse associations between morally injurious outcomes and community involvement, as well as relationship quality [65], and Currier et al., (2017) found a moderate inverse association between perceived social support and morally injurious outcomes [66]. While isolation and challenge engaging in pleasurable activities are listed in Cluster D of the PTSD diagnosis, MI may intensify both of these, and further hamper the social domain of health. This hampering may be most acute when there are aspects of broken trust, as a high level of trust is required to fulfil operational duties. As a result, institutional betrayal may be associated with or compound MI [67,68].

Researchers have theorized that treadmill walking during 3MDR may enhance divergent thinking. It is tempting to speculate that this may facilitate greater cognitive flexibility, allowing for the recognition and processing of problematic thoughts related to morally injurious experiences. Bravo et al., (2020) noted that both self-directed and other-directed MI was associated with increased rumination and problem-focused thoughts, which in turn was associated with higher reported symptoms of depression, anxiety, and PTS [69]. Similarly, Boska & Capron (2021) found that MI was defined by distorted cognitions related to atonement, self-worth and judgment, the reliability and trustworthiness of others, and forgiveness of others, while PTSD was defined by the threat of harm and forgiveness of the situation [70]. Divergent thinking entails the generation of many ideas about and alternative solutions to a problem [71]. Participants in the study were required to experience, identify, and give voice to emotions that were often linked to problematic and ruminative thoughts, narratives (about self and others), and existential questions. In conjunction with moral emotions, some experienced cognitive dissonance concerning themselves, others, or a higher power. Increasing divergent thinking may allow participants to become more open to challenging and non-normative thoughts (i.e., questioning their training), causing a mental shift in thinking patterns regarding traumatic events and pMIEs.

As participants expressed their emotions, it is hypothesized that they were able to engage in meaning-making processes which enabled them to either accept or come to terms with previously unacceptable components of their stories [72]. Currier et al., (2015a) found preliminary evidence that difficulties with meaning-making could serve as a mediating pathway for how pMIEs increase the risk for adjustment problems after warzone service [73]. Kopacz et al., (2019) have also noted that meaning-making includes a reconciliation of appraisals or understandings about specific stressful life events with core beliefs about the self, others, and the world (i.e., their global beliefs) [74]. This may be represented within the Moral Sensemaking theme, as participants reported 3MDR assisting not only with cognitive restructuring, but with repairing damaged global beliefs and worldviews, and re-interpretating meaning, purpose, and intentionality. Moreover, it was not until these actions were completed that healing could begin and some relief from the MI was experienced.

### Strengths and Limitations

This study has several important strengths and limitations. To the best of our knowledge, it is the first study to examine the potential role of MI in combat-related TR-PTSD and whether 3MDR could be an acceptable treatment modality for MI in MMs and veterans. This knowledge may be beneficial for the fields of MI and TR-PTSD, as the understanding may provide new insights into these two emerging and perplexing constructs. Second, as a mixed-method study, it offered a holistic perspective on the participant experience and sought to draw deeply on the qualitative aspects to further illuminate the quantitative data. Third, this study used MI-informed clinicians who may have allowed for the positive results found; this is both a strength and a limitation of the study.

Several important limitations also need to be acknowledged. First, the sample size for this proof-of-concept analysis was small and statistically underpowered largely due to restrictions related to COVID-19; results from the full RCT will be forthcoming. Therefore, caution regarding overgeneralization is warranted for both the quantitative and qualitative data. Second, missing data within the outcome measures may have affected the accuracy of the data analysis. Third, some participants chose not to participate in all of the data collection, resulting in missing longitudinal data. Fourth, it has been widely acknowledged in the literature that standardized questionnaires for MI may be lacking in reliability, validity, and sensitivity [75]. The MISS-M-SF was selected as the best questionnaire at the time of study construction; however, caution is warranted with regard to whether this questionnaire fully captured the causes, symptoms and harm caused by MI. As this area of research is rapidly developing, there may exist other outcome measures for MI that may be appropriate for this population and exhibit improved reliability, validity, sensitivity, and temporal stability. Fifth, given the conceptual challenges associated with MI, it is possible that a thematic analysis may not have fully encapsulated the events and processes that subsequently produced the noted harms associated with MI. Finally, influences such as the fear of judgment around the moral and legal aspects of warfare may have caused participants to limit their self-disclosures, particularly regarding preparation-based acts of MI.

## 5. Conclusions

The results of this proof-of-concept study, while preliminary, showed that not only did MI often co-occur in MMs and veterans with TR-PTSD, but MMs and veterans found 3MDR to be an acceptable treatment for TR-PSTD and MI. As the evidence regarding MI in military and veteran populations continues to evolve, there is a growing imperative that MI be properly assessed and considered in the treatment of combat-related TR-PTSD, PTSD, other comorbid psychiatric disorders, and associated psychosocial challenges. Effectively addressing MI may provide healing and relief from problematic symptoms and impediments to life which are lacking in traditional evidence-based trauma treatments.

## Figures and Tables

**Table 1:** Demographics (n = 11).

Gender	Age	Marital Status	Employment Status	
Female: 1 (9%) Male: 10 (91%)	30–39 years: 2 (18%) 40–49 years: 6 (55%) 50–54 years: 3 (27%) mean: 45.4 ± 6.8 years range: 30.9 to 54.3 years	Common-law: 2 (18%) Divorced: 1 (9%) Married: 5 (45%) Separated: 1 (9%) Single: 2 (18%)	No: 5 (45%) Yes: 6 (55%)	
Military Status	Enrollment Era	Rank	Element	Years of Service
Active: 3 (27%) Veteran: 8 (73%)	1976–1990: 2 (18%) 1991–2000: 8 (73%) 2001–2015: 1 (9%)	Junior NCM: 6 (55%) Senior NCM: 4 (36%) Unknown: 1 (9%)	Air: 2 (18%) Land: 9 (82%)	5–10 years: 2 (18%) 11–15 years: 1 (9%) 20+ years: 8 (73%)

Demographic characteristics are presented vertically, including numbers and percentages (in brackets) of participants falling into the various categories for each characteristic. In the Age column, mean and standard deviation as well as range are also presented. NCM = non-commissioned member. The enrollment era also indicates the years in which the participants enrolled in the CAF and not necessarily the years that the participants served. As noted by the years of service, many participants served multiple years (e.g., a participant enrolled in 2001 may have served until 2011, resulting in multiple deployments).

## Data Availability

Data may be available upon direct request from the authors. Availability will be decided on a case-by-case basis, with the author’s discretion to maximize the anonymity and confidentiality of the participants in this study.
